# Case Report: Minocycline-induced drug reaction with eosinophilia and systemic symptoms syndrome: a case report and literature review

**DOI:** 10.3389/fphar.2024.1355774

**Published:** 2024-03-07

**Authors:** Zhe Zhao, Ming Lu, Yingqiu Ying

**Affiliations:** ^1^ Department of Pharmacy, Peking University Third Hospital, Beijing, China; ^2^ Institute for Drug Evaluation, Peking University Health Science Center, Beijing, China; ^3^ Department of Infectious Disease, Peking University Third Hospital, Beijing, China; ^4^ Department of Respiratory and Critical Care Medicine, Peking University Third Hospital, Beijing, China

**Keywords:** minocycline, drug reaction with eosinophilia and systemic symptoms (DRESS) syndrome, adverse event, case report, literature review

## Abstract

Minocycline is a tetracycline commonly used for several dermatological diseases. Drug reaction with eosinophilia and systemic symptoms (DRESS) syndrome is a rare but severe adverse event which can be caused by minocycline. An 18-year-old male patient developed fever, acute rash, pharyngeal pain, lymphadenopathy, hematologic abnormalities, increased creatinine level, elevated liver enzyme levels, and splenomegaly 4 weeks after the oral treatment of minocycline, 100 mg daily, for acne. Once diagnosed with DRESS syndrome, intravenous methylprednisolone was applied and his clinical manifestations and laboratory results remarkably improved. Then, a total of 13 DRESS syndrome cases induced by minocycline were reviewed and their clinical characteristics were summarized. In these cases, only two patient (15.4%) was present with pharynx involved. In conclusion, we reported a rare minocycline-induced DRESS syndrome who developed fever, eosinophilia, acute rash, pharyngitis, lymphadenopathy, acute kidney injury, hepatitis, and splenomegaly. Our report provides detailed clinical features of minocycline-induced DRESS syndrome, which helps us further understand this severe adverse event.

## 1 Introduction

Minocycline is a semisynthetic tetracycline widely used for treatment of several dermatological diseases, like acne, rosacea, and bullous dermatoses, due to its good antibiotic ability, anti-inflammatory activity, and skin penetration (Eichenfield et al., 2021). However, its systemic usage is associated with some severe autoimmune adverse events, including drug-induced lupus, idiopathic intracranial hypertension, and drug reaction with eosinophilia and systemic symptoms (DRESS) syndrome (Hung and Chung, 2022). According to a systematic review, 3.3% (41/1,230) of the patients with acne vulgaris treated with minocycline had symptoms suggesting an acute hypersensitivity reaction (Garner et al., 2000). The incidence of DRESS syndrome is estimated to be more than 1 case in 10,000 exposures to medications in general population (Calle et al., 2023). The Registry of Severe Cutaneous Adverse Reactions (RegiSCAR) study shows that DRESS syndrome affects patients at a mean age of 47.4 years (Fernando, 2014). Here, we report a case of a young man who developed fever, eosinophilia, acute rash, pharyngitis, lymphadenopathy, acute kidney injury (AKI), hepatitis, and splenomegaly after the 4-week use of minocycline for acne treatment, which was successfully controlled by steroid pulse therapy.

## 2 Case presentation

An 18-year-old male was admitted to the hospital with a 3-day history of fever and rash. Four weeks ago, he was diagnosed with acne and received the treatment with minocycline, 100 mg daily, for 3 weeks. His other medical history was unremarkable.

**FIGURE 1:**
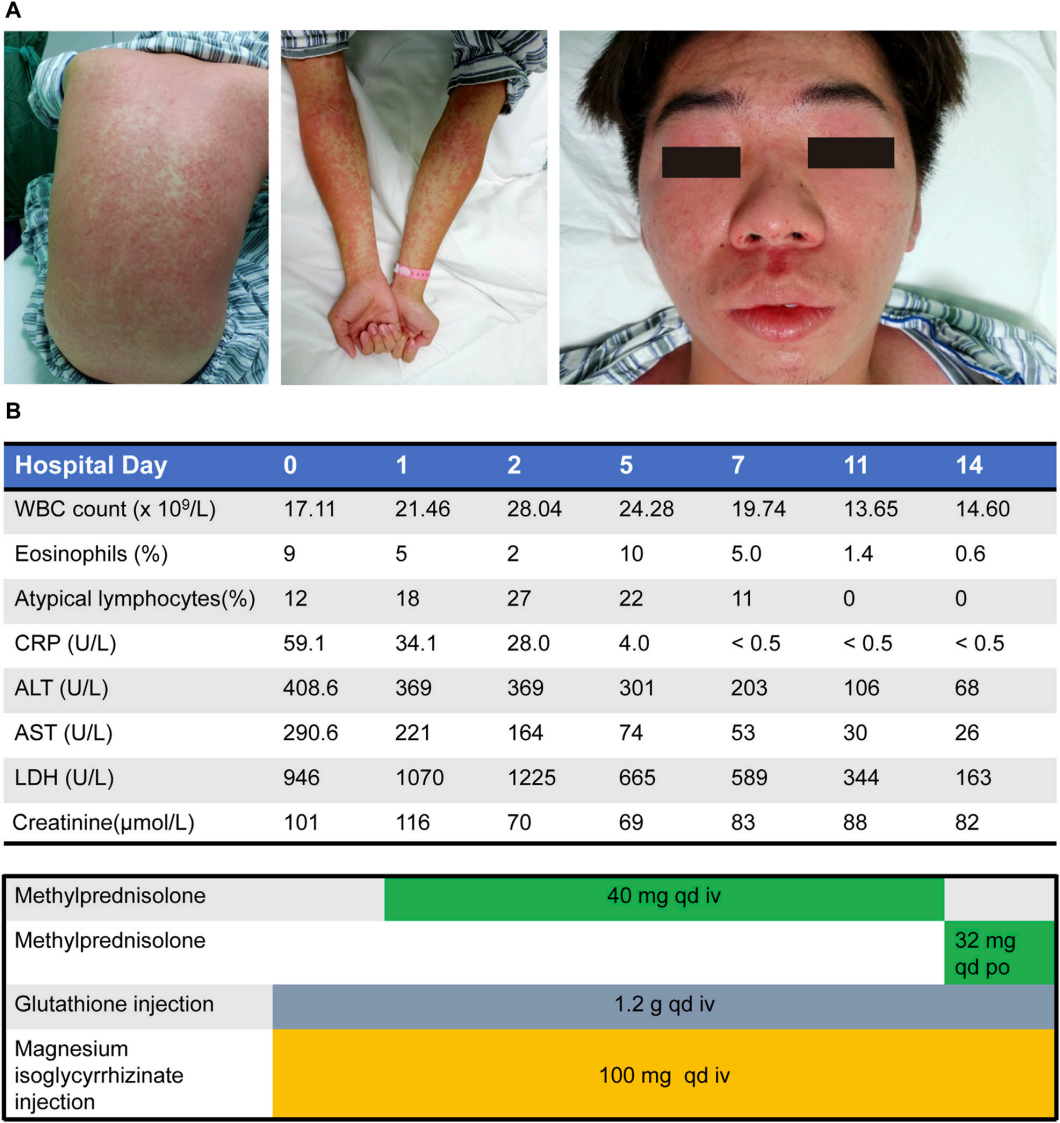
Minocycline-induced drug reaction with eosinophilia and systemic symptoms. **(A)** Diffuse erythematous or maculopapular eruption spread most of the entire body and the presence of facial edema. **(B)** Clinical course of drug administration and laboratory results during the hospitalization. ALT, alanine aminotransferase; AST, aspartate aminotransferase; CRP, C-reactive protein; iv, intravenous; LDH, lactate dehydrogenase; po, peros (oral); qd, quaque die (daily); WBC, white blood cell.

On the initial presentation after admission, he had a diffuse erythematous or maculopapular eruption with pruritus, from the anterior chest spreading to the trunk and extremities. Facial swelling was also found (Figure 1A and Supplementary Figure S1). Intermittent fever peaking at 39.9°C, cough, and pharyngeal pain developed. His pulse rate measured 110 beats per minute, and blood pressure was recorded at 118/76 mmHg. Physical examination revealed diffuse red papules on the face, trunk, extremities, swelling of the lips and scattered ulcers visible on the oral mucosa. Multiple lymphadenopathies involved bilateral cervical, axillary, and inguinal nodes. The lungs are clear to auscultation bilaterally, without any wheezes, rales, or rhonchi.

The laboratory test showed an increased WBC count of 17.11 × 10^9^/L with 9% eosinophils and 12% atypical lymphocytes. Besides, no other hematologic abnormalities were identified in our case. His serum creatinine level was increased at 116 μmol/L and urine protein was positive. His liver enzyme levels were abnormal, including elevated alanine aminotransferase (ALT) level at 369 U/L, aspartate aminotransferase (AST) level at 221 U/L, and lactate dehydrogenase (LDH) level at 1070 U/L. The bilirubin levels were within normal ranges. His blood ferritin level was elevated at 1,223 ng/mL, C-reactive protein was increased at 34.4 mg/L, and procalcitonin level was 1.3 ng/mL (Figure 1B). Besides, his immunoglobulin G and immunoglobulin M were decreased at 6.54 g/L and 0.39 g/L, respectively, and the serum complement level were decreased (C3 at 0.738 g/L). Meanwhile, the detected autoantibodies, including anti-nuclear antibody (ANA), anti–double-stranded DNA antibody, and anti-smooth muscle antibody (SMA) were all negative. Abdominal ultrasound test revealed splenomegaly. Although the creatinine kinase (CK) level was elevated at 222 U/L and CK-MB was slightly elevated at 30.1 U/L, his serum troponin levels, electrocardiogram (ECG) and transthoracic echocardiography (TTE) were normal, suggesting a low possibility of myocarditis. Chest CT images revealed no infiltration in both lungs. These results together suggested that skin, pharynx, lymph nodes, kidney, liver, and spleen were involved. Other laboratory tests found no evidence for any viral infections related to DRESS syndrome, including measles and rubella viruses, Epstein-Barr virus (EBV), cytomegalovirus (CMV), hepatitis viruses, and human herpes virus 6 or 7 (HHV-6/7).

Considering the acute rash, increased WBC count, eosinophilia, fever, and systemic involvement, this patient was diagnosed with DRESS syndrome. Minocycline was promptly discontinued and intravenous methylprednisolone (40 mg daily) was administrated for 11 days. Then, oral treatment of methylprednisolone (32 mg daily) was used instead. In addition, the intravenous administration of glutathione (1.2 g daily) and Mg isoglycyrrhizinate (100 mg daily) were prescribed for 2 weeks to improve the liver function. The skin eruption in the trunk and extremities and facial edema improved remarkably (Supplementary Figure S1), and his WBC count, kidney and liver function returned to normal (Figure 1B). Then, methylprednisolone was maintained for 3 weeks and tapered gradually over the following 2–3 months. During the follow-up of 6 months, no recurrence of fever and rash occurred. Written consent for publication was obtained from the patient.

## 3 Discussion

DRESS syndrome is a rare but severe drug hypersensitivity reaction featured by a delayed onset, variable clinical symptoms, and a prolonged course. The actual incidence of DRESS syndrome is diverse depending on the medication prescribed and the immune status of every patient. In the general population, the incidence is estimated to be more than 1 case in 10,000 exposures to medications (Calle et al., 2023). DRESS syndrome symptoms usually appear 2 weeks to 2 months after initial treatment (Hung and Chung, 2022). The diagnostic criteria of DRESS syndrome include the suspicion of a drug-related reaction as well as the occurrence of acute rash, fever (>38°C), lymphadenopathy, hematologic abnormalities, and systemic involvement (Martins et al., 2021). The systemic involvement of internal organs may manifest as hepatitis, nephrotoxicity, pneumonitis, cerebral edema, eosinophilia, pericardial effusion, leukocytosis, myocarditis, or thyroiditis (Taweesedt et al., 2019; Radovanovic et al., 2022; Dagnon da Silva et al., 2023). In our case, the patient had pharyngitis, lymphadenopathy, AKI, hepatitis, and splenomegaly. His AKI manifested as increased creatinine and proteinuria. A recent systematic review summarized 71 cases with renal manifestations of DRESS syndrome showed that AKI was the most common manifestation in 96% of the patients. 27% had AKI with proteinuria and anuria was the rarest manifestation (Dagnon da Silva et al., 2023). The mortality rate associated with DRESS syndrome is 10%–20% usually due to myocarditis or hepatotoxicity (Heymann, 2020). Common drugs that may cause DRESS syndrome include aromatic anticonvulsants, sulfonamides, salazosulfapyridine, allopurinol, calcium blockers, terbinafine and minocycline (Hama et al., 2022). Emerging evidence indicates a correlation between specific medications and organ involvement in DRESS syndrome. Allopurinol has been frequently linked to renal manifestations, while minocycline is often associated with cardiac or pulmonary manifestations in DRESS syndrome (Taweesedt et al., 2019). In this case, an assessment using Naranjo Adverse Drug Reaction Probability Scale showed a score of 6 (Naranjo et al., 1981), suggesting his DRESS syndrome was a probable adverse event related to minocycline.

Minocycline is a secondary-generation tetracycline agent with a broad spectrum of activity. It effectively targets a wide range of bacteria, especially the drug resistant ones like community-acquired methicillin-resistant *S. aureus*. Minocycline is commonly prescribed for the treatment of acne vulgaris owing to its high antimicrobial activity against *Propionibacterium acnes* and good skin penetration (Eichenfield et al., 2021). In our case, minocycline was used for the treatment of acne vulgaris. However, more frequent or severe adverse events have been found to be associated with the oral usage of minocycline than other tetracyclines, which was estimated to occur in 13.6% of all minocycline-treated patients (Patel and Bhatia, 2021). Common adverse events related to systemic minocycline can affect the nervous (vertigo, headache), gastrointestinal (nausea, vomits, diarrhea), musculoskeletal (myalgia, arthritis), respiratory (dyspnea), and cutaneous (hyperpigmentation, urticaria, rash, pruritus) systems (Martins et al., 2021). Meanwhile, DRESS syndrome is a very rare adverse event associated with minocycline usage.

For now, only some cases of minocycline-induced DRESS syndrome have been reported, but no systematic reviews have been conducted to summarize the clinical features of DRESS syndrome caused by minocycline. To perform a thorough literature review, we searched PubMed and Embase databases by using “Drug reaction with eosinophilia and systemic symptoms”, “DRESS syndrome”, “drug-induced hypersensitivity reaction”, “DIHS”, “Drug Hypersensitivity Syndrome”, or “DHS”, and “minocycline” as keywords. After screening and assessment, a total of 13 eligible cases were included for analysis (Tsuruta et al., 2006; Brown et al., 2009; Eshki et al., 2009; Shaughnessy et al., 2010; Taylor et al., 2012; Kanno et al., 2014; Wu and Anadkat, 2014; Lan et al., 2015; Kirchhof et al., 2016; Gowani et al., 2018; Kudlaty et al., 2023; Shea et al., 2023; Zhao et al., 2023) (Supplementary Figure S2). The clinical characteristics of these cases were summarized and demonstrated in Table 1. The age of patients in these cases ranged from 13 to 62 years, nearly half of them (6/13) were under 18 years 84.6% of these patients (11/13) were female. Most of the patients were prescribed minocycline for the treatment of acne or folliculitis, while in some other cases minocycline was used for cystitis, perioral dermatitis, central centrifugal cicatricial alopecia or confluent and reticulated papillomatosis. Only 3 cases provided the dosage of minocycline, with two patients receiving a dosage of 100 mg (Brown et al., 2009; Zhao et al., 2023) and one patient receiving a dosage of 150 mg (Tsuruta et al., 2006). Ten patients developed DRESS syndrome after 2–5 weeks of oral minocycline administration, and 3 cases demonstrated latency periods of 8, 12 or 15 weeks. Most of the cases developed widespread erythematous eruption of various manifestations. Almost all the patients had lymph nodes and liver involved, but in few cases pancreatic islets (2/13, 15.4%), pharynx (2/13, 15.4%), lung (2/13, 15.4%) were affected. In our case, the patient was present with skin, pharynx, lymph nodes, kidney, liver, and spleen involved, which is a rare minocycline-induced DRESS syndrome case. As shown in Table 1, apart from methylprednisolone initially applied in most cases, cyclosporine, liver transplantation, and prednisone were used for management of DRESS syndrome in other cases. Of the 12 patients with available outcomes, 7 recovered from the adverse event, 1 experienced a second hospitalization, but 4 died of this severe syndrome.

**TABLE 1:** Different cases of DRESS syndrome associated with minocycline administration.

Author, year	Age/sex	Indications	Latency period (week)	Rash morphology	Peak temperature	Organ involvement	HHV	Management	Outcome
Shea et al	16/M	Acne	4	Erythematous papules and plaques, morbilliform eruption	NR	Bone marrow	NR	Methylprednisolone 1 mg/kg/day × 3 days, then 60 mg/day × 7 weeks	Recovery
Kudalaty et al	62/F	Central centrifugal cicatricial alopecia	3	Erythematous facial rash	39	Lymph node, liver, CNS	HHV-6/7 positive	Methylprednisolone 60 mg every 8 h	Recovery
Shaughnessy et al	38/F	Acne	3	Erythematous eruption	38.4	Lymph node, liver, myocardium	NR	Methylprednisolone 1 mg/kg/day	Second hospitalization
Kirchhof et al	30/M	Folliculitis	2	Erythematous edematous papules coalescing into plaques	38.2	Lymph node, liver, lung	NR	Cyclosporine 5 mg/kg/day	Recovery
Gowani et al	18/F	Folliculitis	5	Macular morbilliform rash	NR	Lymph node, liver	HHV-6 negative	Prednisone	Recovery
Kanno et al	60/F	Cystitis	12	Erythematous eruption, excoriations and desquamation	38.1	Lymph node, liver, myocardium	HHV-6 positive	Methylprednisolone 1 g/day × 3 days, prednisolone 1 mg/kg/day	Recovery
Eshki et al	15/F	Acne	15	Maculopapular exanthema	40	Lymph node, liver, pharynx	HHV-6 positive	Methylprednisolone, oral prednisolone 1 mg/kg/day	Death
Zhao et al	14/F	Confluent and reticulated papillomatosis	4	Pseudovesicular erythematous papules	NR	Lymph node, liver, lung, kidney	NR	Methylprednisolone 1 mg/kg/day × 3 days, dexamethasone, anakinra, siltuximab, ruxolitinib	Death
Wu et al	46/F	Perioral dermatitis	3	Scaly erythematous eruption	39.5	Lymph node, liver, myocardium, kidney	NR	Methylprednisolone 40 mg/day	Death
Lan et al	13/F	Acne	3	NR	41	Liver, kidney, pancreatic islets	HHV-6 positive	High-dose steroids, liver transplantation	NR
Taylor et al	16/F	Acne	3	Urticaria, maculopapular rash	NR	Lymph node, liver, myocardium, kidney	NR	Prednisone	Death
Brown et al. (2009)	15/F	Acne	4	Erythematous eruption, progressive erythroderma	NR	Liver, pharynx, pancreatic islets, thyroid, lymph node	NR	Prednisone 10 mg/day x 4 days, then corticosteroid x 4 months	Recovery
Tsuruta et al. (2006)	20/F	Acne	8	Pruritic, exudative maculopapules	40	Liver, lymph node	HHV-6 negative	Prednisone 1 mg/kg/day × 3 days, then 0.6 mg/kg/day × 4 days, then 0.3 mg/kg/day × 7 days, then 0.2 mg/kg/day × 7 days	Recovery

CNS, central nervous system; HHV, human herpes virus; NR, not reported.

The pathogenesis of minocycline-induced DRESS syndrome remains unclear, but some hypotheses have been proposed. Since minocycline is metabolized in liver by CYP3A4, its abnormal activity has been found to play a critical role in the development of DRESS syndrome (Martins et al., 2021). The accumulation of reactive drug metabolites caused by the deficient detoxification enzyme activity may interact with proteins to induce autoimmune responses. Clinicians should be mindful of potential drug interactions when prescribing minocycline in combination with other medications affecting CYP3A4 activity, since these drugs may influence the clearance of minocycline, potentially inducing DRESS syndrome (Martins et al., 2021). In addition, genetic polymorphisms of CYP3A4 should also be taken into consideration. In a series of African cases, the persistence of minocycline in the skin or plasma was associated with the relatively higher frequency of a mutant genotype for a detoxification enzyme. The prolonged DRESS syndrome may be explained by the formation of a melanin-minocycline complex (Maubec et al., 2008). Besides, increasing evidence has indicated that viral infection is another potential contributor of DRESS syndrome, like HHV-6/7, CMV, and EBV. This theory proposes that these viruses may trigger the proliferation of T cells to produce cytokines, which may lead to the hypersensitivity reactions (Hama et al., 2022; Pichler and Brüggen, 2023). In our literature review, 5 cases were tested for HHV infection and 4 of them showed positive HHV-6, suggesting the potential role of HHV reactivation in the pathogenesis of minocycline-induced DRESS syndrome (Table 1). To be noted, our patient had negative results for all these viral infections, indicating some other mechanisms may contribute to his DRESS syndrome.

For the quick recovery and positive prognosis in this case, early diagnosis and prompt management are both predominant. Firstly, DRESS syndrome can be identified according to its characteristic cutaneous manifestations. Then, systematic evaluation should be performed and the RegiSCAR scoring system is an approach most commonly used for diagnosis (Calle et al., 2023). According to the scoring system, our case scored 8, falling into the category of “definite” DRESS syndrome. In addition, it is imperative to rule out autoimmune diseases including systemic lupus erythematosus (SLE) and autoimmune hepatitis. However, given the negative results for ANA, anti-dsDNA and SMA and decreased IgG in this case, a diagnosis of SLE or autoimmune hepatitis is unlikely. Once diagnosed, the suspected culprit medication should be immediately withdrawn. Unlike this case, determining the causative drug can be challenging in some cases. If so, clinical tests like skin patch tests and lymphocyte transformation tests are usually recommended (de Groot, 2022). In addition, systemic administration of corticosteroids should be promptly initiated. Besides, upon recognizing the liver dysfunction on hospitalization, glutathione and Mg isoglycyrrhizinate were applied. These two drugs act as antioxidants and possess anti-inflammatory properties, which have been widely used to protect the liver in China (Fan et al., 2022). Owing to the prompt treatment, his liver function recovered soon. However, it should be noted that high-quality randomized clinical trials supporting the use of these two drugs in treating liver failure are still limited, warranting further investigation. With timely management, most patients can fully recover from DRESS syndrome. This report does bring to mind that dermatologists should be aware of this severe adverse reaction during the systemic administration of minocycline for acne.

In conclusion, we here report a case of minocycline-induced DRESS syndrome characterized by fever, eosinophilia, acute rash, pharyngitis, lymphadenopathy, AKI, hepatitis, and splenomegaly. It is a rare but serious adverse event that need early diagnosis and prompt treatment. Since minocycline is a widely used drug for treatment of dermatologic diseases, dermatologists should be aware of this severe adverse reaction before the systemic administration of minocycline.

## Data Availability

The original contributions presented in the study are included in the article/Supplementary Material, further inquiries can be directed to the corresponding authors.
