# Supplementary material for: Case Report: Minocycline-induced drug reaction with eosinophilia and systemic symptoms syndrome: a case report and literature review
Source: Front Pharmacol. 2024 Mar 7;15:1355774. doi: 10.3389/fphar.2024.1355774 (PMC10955048; doi:10.3389/fphar.2024.1355774)
Supplement: Supplementary file 1 [file Table1.DOCX]

Supplementary Material

# Supplementary Figures and Tables

## Supplementary Figures

**
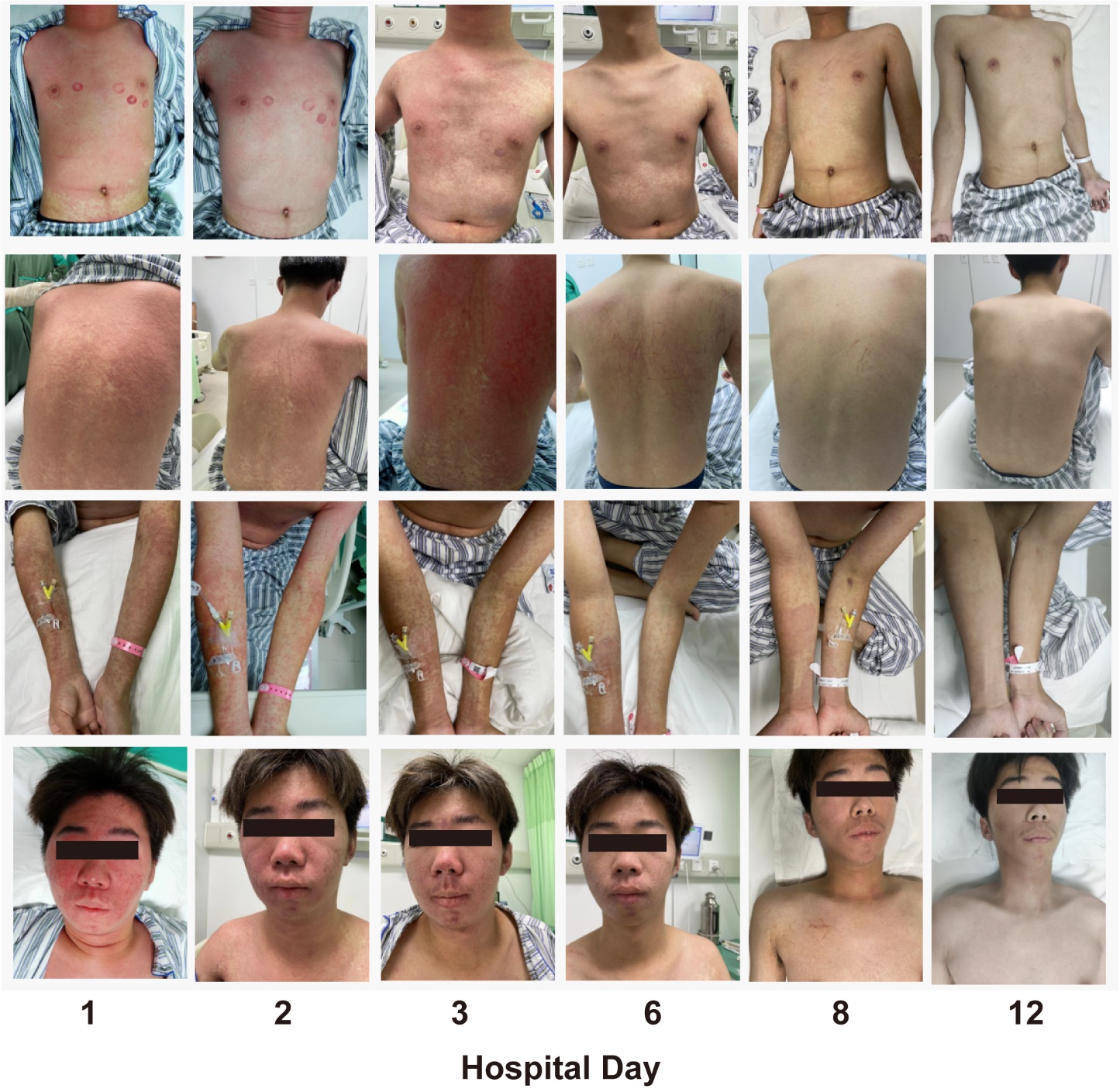
**

**Supplementary Figure 1.** Improvement of skin eruption and facial edema during the hospitalization.





**Supplementary Figure 2.** Flowchart of literature review.
